# Patient participation in research funding: an overview of when, why and how amongst Dutch health funds

**DOI:** 10.1186/s40900-019-0163-1

**Published:** 2019-11-11

**Authors:** Willemijn M. den Oudendammer, Jacquelien Noordhoek, Rebecca Y. Abma-Schouten, Lieke van Houtum, Jacqueline E. W. Broerse, Christine W. M. Dedding

**Affiliations:** 1 Vrije Universiteit Amsterdam, de Boelelaan 1085, 1081 HV Amsterdam, The Netherlands; 2 Nederlandse Cystic Fibrosis Stichting, Dr. A Schweitzerweg 3, 3744 MG Baarn, The Netherlands; 3 Nederlandse Hartstichting, Prinses Catharina-Amaliastraat 10, 2496 XD Den Haag, The Netherlands; 4 Nederlands Diabetes Fonds, Stationsplein 139, 3818 LE Amersfoort, The Netherlands; 5 Medical Humanities, Amsterdam UMC, Vrije Universiteit Amsterdam, de Boelelaan 1089a, 1081 HV Amsterdam, The Netherlands

**Keywords:** Patient participation, Patient involvement, Patient inclusion, Shared decision-making, Health-related research funding, Research-funding agencies, Health funds, Research funding process

## Abstract

**Background:**

Patient participation in decision-making on health-related research has gained ground. Nineteen Dutch health-related research-funding organisations (HFs) have taken up the challenge to include patients in their funding process. A ‘Patient participation (PP) advisory team’ was set-up, with HF-representatives and patient advocates, who together initiated this study. We provide an overview of *when*, *why*, and *how* PP activities take place in HFs’ funding processes, share main challenges and identify possible solutions.

**Methods:**

A qualitative research design was used. Data was gathered by questionnaires (*n* = 14) and semi-structured interviews (*n* = 18) with HF employees responsible for patient participation, followed by a workshop (*n* = 27) with involved employees of HFs and key players in PP from national patient organisations and research organisations. A descriptive analysis was used for the questionnaire. A semi-directed content analysis was used for the interviews and the workshop.

**Results:**

Three stages can be identified in the funding process in which HFs carry out PP activities: (1) strategic decision-making about focus of research (e.g. shared research agendas); (2) call for and receipt of research proposals (e.g. mandatory inclusion of letter of recommendation from patient organisation); (3) decision-making about the funding of research proposals (e.g. patients reside in a patient panel to co-review research proposals). Main challenges identified to carry out PP activities include: how to accommodate diversity of the patient body (mainly encountered in stage 1 and 3); to what extent should patients receive training to successfully participate (mainly encountered in stage 1 and 3); and who is responsible for patient-researcher dialogues (mainly encountered in stage 1 and 2). All nineteen HFs agree that patients should be included in at least one stage of the funding process for health-related research. CONCLUSION: Further broadening and optimising patient involvement is still needed. The proposed solutions to the identified challenges could serve as inspiration for national and international research funding foundations that aim to structurally include patients in their funding process.

## Plain English summary

Health research is important for patients: it helps to discover new ways to diagnose disease, to develop effective therapies and to improve quality of life. Dutch research funding organisations increasingly want to involve patients in deciding which research to fund, each finding their own way in how to work with patients in a meaningful way. This is not an easy process. Therefore they, together with patient advocates, asked for an overview of *when*, *why*, and *how* patients are currently involved in these decisions and how this process could be optimised. We gathered information with questionnaires, organised a group discussion with patient advocates, patient organisations, health foundations and interviewed employees of the different health foundations. We discovered that *all* foundations include patients in their decisions, but they do it in different ways, at different stages, and not at the same depth. Nevertheless, they all face the same challenges: they find it difficult to find patients from different sociocultural backgrounds; they find it difficult to decide if and what training they should provide for patients in order to become participants/partners; and they feel that not all researchers are open to, and take up the responsibility to actually involve patients in their research. We conclude that more cooperation and (inter) national exchange of knowledge is needed, to address the challenges and we provide some first ideas to start with.

## Background

Patient participation (PP) initiatives in the research process have been appearing since the 1970s, for example the founding of a National Association for Patient Participation in the UK and a testimony written by an American patient regarding her disease which led to her reviewing research proposals for a funding organisation for cancer research [1, 2]. It is however only recently that PP has gained ground and become more widely accepted [3–5]. Previously, decision-making on health-related research was mainly dominated by experts such as medical doctors, researchers and policy-makers [6–8]. Patients were invited to provide information on living with a disease and issues related to quality of life. Since then, PP has evolved, and patients are increasingly engaged in decision-making on research, initially in setting research agendas [9, 10] and more recently in appraising research proposals [5]. In this study we use ‘patient participation’, (in literature) this is also known as patient involvement or engagement.

The new role of patients is characterised as a transition from a supply-driven towards a demand-driven health research system [11, 12]. Such a transition is considered necessary, not only from a normative perspective (patients have the right to be involved in decisions affecting their lives), but also to increase the social impact of research [13, 14].

Research-funding organisations are often mentioned as actors in the health research system which could take up a role as change agent, facilitating the transition process [3, 4, 8]; they have the power to pressurise researchers to involve patients in their projects, while they can engage patients in their own funding procedures. By taking up this role, more effective patient participation can take place. This is unlikely to be an easy or straightforward process, given the prominent position of researchers and medical doctors on the advisory committees of research-funding organisations. Nevertheless, several research-funding organisations have taken up the challenge to include patients in their working procedures, including the UK and the Netherlands.

Previous research sets the stage for patient participation in research in the UK and the Netherlands and shows that patients’ experiential knowledge can contribute to the relevance and quality of health-related research [6]. The majority of the Dutch private and public, non-profit, research-funding organisations are disease-specific health funds (HFs) that together support a significant proportion of health-related research in the Netherlands. Several of these HFs are also a patient organisation, others have an official partnership with a patient organisation or work together project-based without an official partnership. Nineteen HFs (at time of research) had joined forces in the umbrella organisation Collaborative Health Funds (Samenwerkende Gezondheidsfondsen, *SGF*). Their overall goal is to positively impact the lives of (future) patients. Their core business is the financing of scientific research; in 2016 they contributed for about 200 million euro in scientific research in the Netherlands. Financial support comes from approximately 3 million donors and about half a million volunteers support the HFs in their fundraising activities. Under the SGF the HFs work together on mutually relevant themes, such as PP in research, policy and care [15]. PP in research is defined by the SGF as *giving experiential knowledge an optimal place in order to influence research.* HFs carry out several PP activities in different stages of their research funding decision-making process. There are differences in how long HFs have been working on PP and also in the time, money and manpower allocated to it; PP may be structurally incorporated, project-based or in a pilot phase. The Dutch HFs who are member of the SGF want to collaborate on strengthening patient participation in their research funding decisions, leading to the formation of a PP advisory team. The advisory team consists of 10 HF representatives who are responsible for PP in the activities of their HF or are interested in doing so, furthermore a patient advocate is part of the team as an advisory member. The PP advisory team initiated this study on PP amongst the HFs, based on, amongst other things, advice given by patient organisations on giving patients a serious voice in health-related research decisions [16].

Unfortunately, there are few scientific studies on patient involvement in research-funding decisions. As a consequence, organisations cannot learn substantially/systematically from the experiences of others. At the same time, the literature shows that concerns have been expressed regarding tokenism (mere symbolic participation to appear inclusive). For example, in a UK-based study, funding bodies found that sometimes PP was merely a ‘tick-the-box’ exercise [8]. Some studies address these issues, most often in a specific setting, such as PP in funding decisions for specific diseases [3–5]; papers written from a patient organisation’s point of view [16]; and based on national views ([17]; van [8]).

**Fig. 1:**
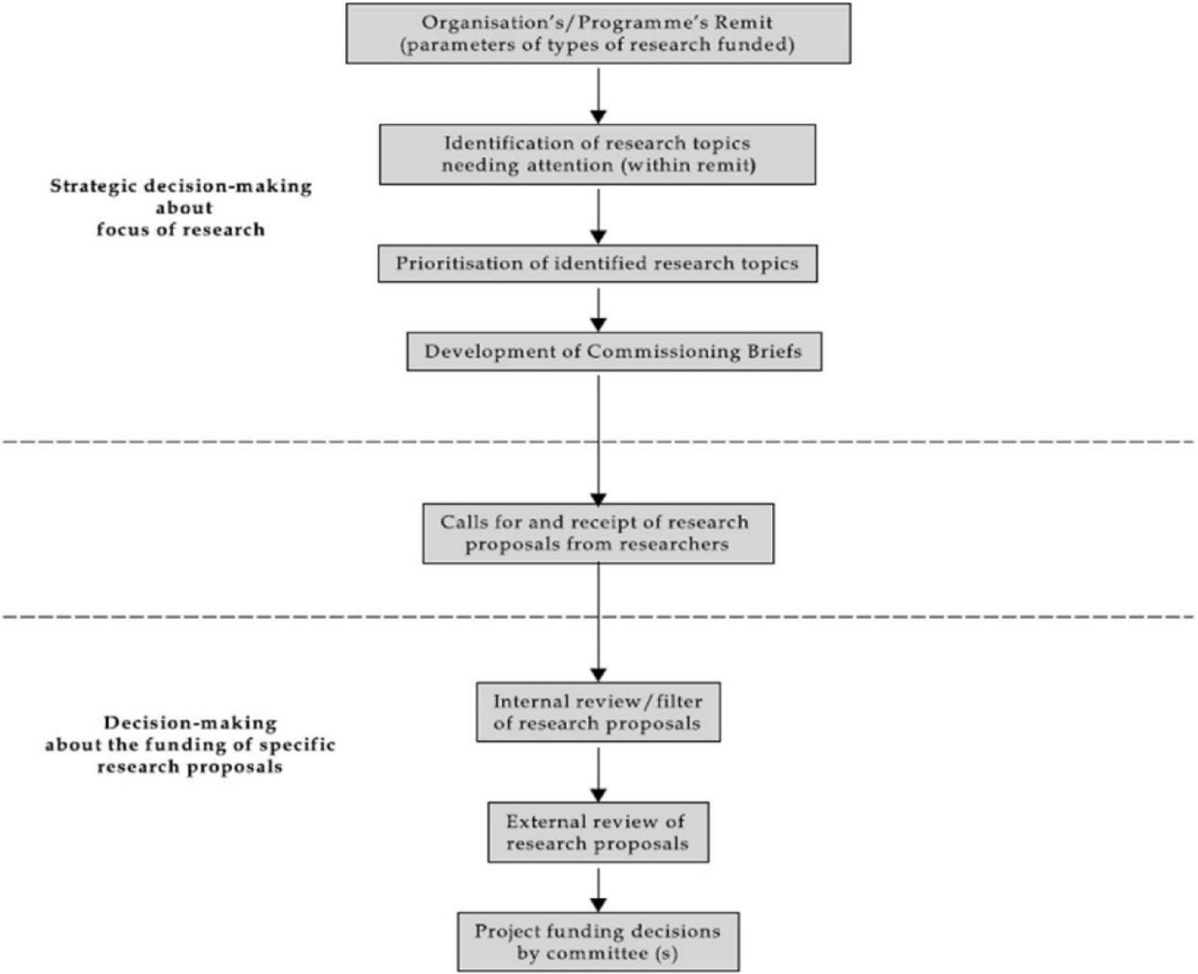
Stages in decision-making regarding research funding in the UK [18]

We use the flowchart presented in O’Donnell and Entwistle’s study to provide an overview of *when*, *why* and *how* PP activities take place by the Dutch HFs; we share the main challenges experienced by the HFs and identify possible solutions and directions for the future. The flowchart shows how research-funding foundations involve consumers in funding decisions of health-related research in the UK [18]. Consumers included “patients, carers, long-term and potential users of health services, and organisations representing consumers’ interests” (p.282). Involvement meant “some form of active partnership between consumers and researchers in the research process, rather than the use of consumers as ‘subjects’ of research” (p.282). They developed a flowchart which identified three basic stages within the research funding decision-making process (henceforth: funding process) of funding organisations in the UK: (stage 1) strategic decision-making about the focus of research, (stage 2) calls for and receipt of research proposals from researchers, and (stage 3) review of research proposals and research funding decision-making (Fig. 1).

## Methods

A qualitative research design was used, including (1) secondary data analysis of a questionnaire set out amongst the 19 HFs (14 responders), (2) semi-structured interviews with HF representatives (*n* = 18), and then (3) a workshop with representatives of HFs, patient organisations and research organisations (*n* = 27).

### Questionnaire

A questionnaire was developed by the PP advisory team of the SGF and distributed among all its members, before the current study was set-up. Employees responsible for PP from fourteen out of nineteen HFs completed the questionnaire. It asked about embedding PP activities within the HF; how the HF stimulated PP; the challenges encountered; future plans regarding PP; and whether or not the HF desired to work together with other HFs or other (patient) organisations on PP. The data were collected during 2014 and primary analysis took place in order to answer questions regarding current PP activities.. We used the results of this questionnaire as secondary data, outcomes of analysis were used as a starting point for the semi-structured interviews.

### Semi-structured interviews

Between November 2015 and March 2016, semi-structured interviews were held with employees of 18 of the 19 SGF members. The remaining HF was not interviewed because it stated that no changes had taken place within its organisation since the questionnaire. To prevent bias, the interviews were held by a researcher not affiliated to one of the HFs. All interviewees were involved in PP activities in their HF. The HFs differ greatly in size and in the manpower, financial resources and time allocated to PP. The interviewees therefore differed as well, ranging from CEOs and managers of research departments to policy officers of PP teams.

The goals of the semi-structured interviews were to assess: (1) whether and why PP activities take place within the specific HF; 2) when and which PP activities are carried out; and 3) what challenges or concerns are experienced. The interviews were conducted by telephone and lasted 20–60 min. Interviewees were made aware that participation was voluntary, and personal statements and individual HFs would not be identifiable when reporting the results. All interviews were audio-taped, summarised by two researchers, and later discussed and validated during the workshop.

### Workshop

After all interviews were complete, a half day workshop was organised in April 2016 for all interviewees as well as other employees of the Dutch HFs and key players in PP from national patient organisations and research organisations. In total, 27 people participated. The main challenges and concerns regarding PP in the funding process expressed during the interviews were discussed in small groups. The importance of these topics was first checked with the participants to validate the findings from the interviews, and any missing challenges or concerns were added. Then the main goal of the meeting was addressed: to come up with new ideas to take on these challenges together.

### Ethical approval

This study is deemed ‘non-invasive’ according to Dutch law and therefore did not require approval from a formal medical ethical committee. The researchers adhered to the national Code of Ethics for Research in the Social and Behavioural Sciences involving Human Participants [19].

### Data analysis

A descriptive analysis was used to interpret the data from the questionnaire. A semi-directed content analysis was used for the interviews and the workshop: pre-determined codes were used based on the model of O’Donnell and Entwistle, while also open coding was carried out for a part of the analysis. Two researchers grouped the main findings under the different stages of the research funding decision-making process, by using the pre-determined codes ‘stage 1, 2, 3’. Using open coding, the challenges experienced and concerns expressed during the interviews were listed, and similar ones grouped under overarching themes. They were presented to the PP advisory team for validation and additional concerns could be added if necessary. No new concerns came up.

## Results

**Fig. 2:**
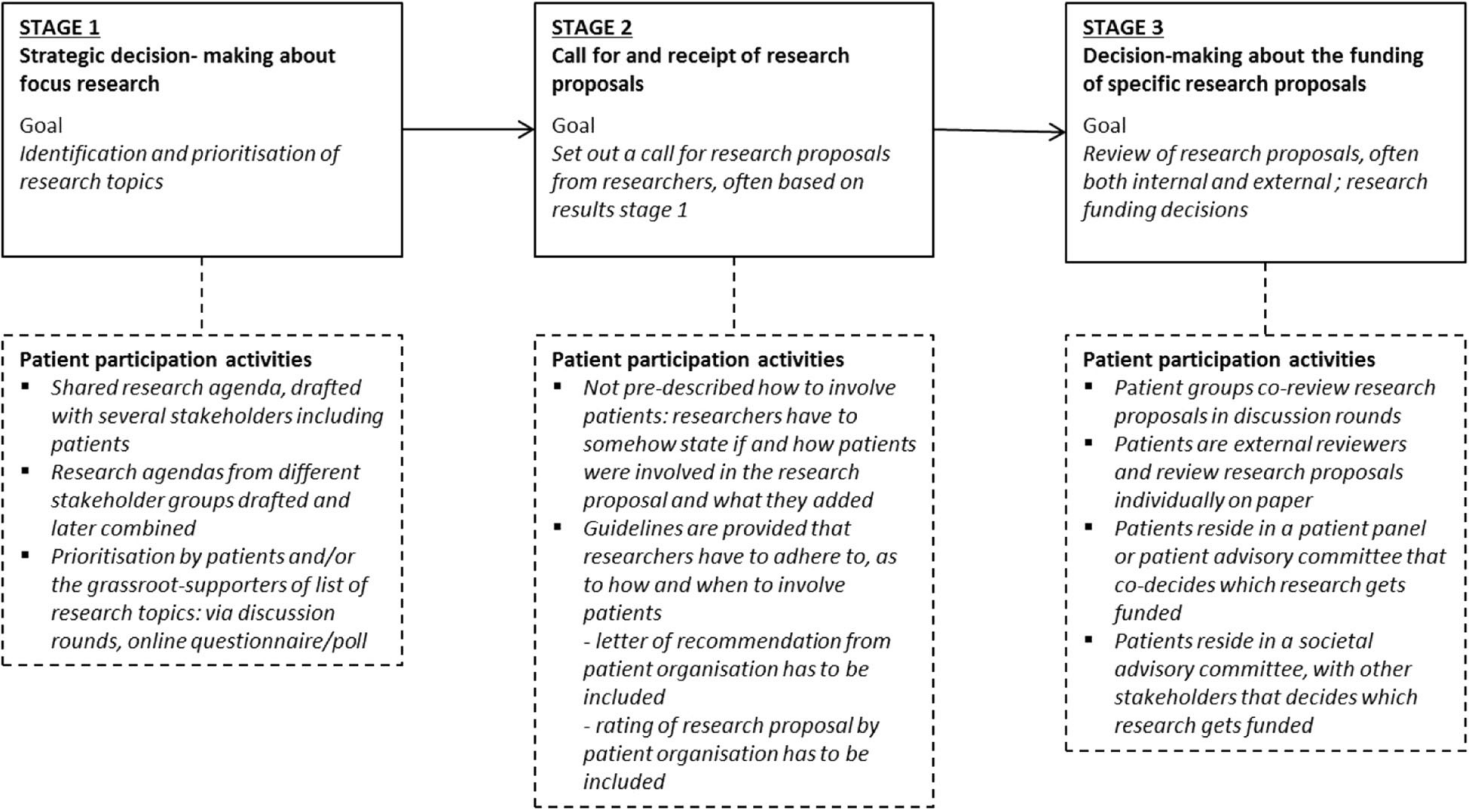
HFs’ PP activities in the stages of the funding process

The results are structured according to the three stages of the funding process defined by O’Donnell and Entwistle (Fig. 2): in stage 1, research topics that need attention are identified and prioritised; in stage 2, a call for research proposals is set out, often specifically for the research themes identified in stage 1, and research proposals are submitted; in stage 3, the research proposals received are reviewed, and final funding decisions are made.

### HFs’ general views on the importance of PP in funding decisions

All 19 HFs acknowledge the importance of PP in one or more stages of their funding process. However, the reasons why they consider PP to be of importance differed somewhat. Three main reasons were given.

First of all, the *‘practice what you preach’* argument. Most HFs want researchers to involve patients in research (proposals); HFs believe they have an exemplary role, and therefore their own actions should convey their ideology.

Second, the idea of *‘for patients, with patients’* was voiced several times. The overall goal of the HFs is to help their target population. This means that HFs work *for* patients and therefore should also be able to *understand* patients by working *with* patients. PP can help clarify what is important from a patient’s view, which will increase the HF’s understanding of the world of their target population and may lead to better decisions. This would improve the quality of research, for example because research will better fit to the needs of patients. PP in some cases may also include the participation of family caregivers or citizens, in which case this is specified.

Third, several HFs mentioned that PP is good for their *legitimacy and transparency towards society*. Many HFs rely on charitable donations from individuals. HFs have experienced that they can better justify their funding decisions to these donors when they work together with patients.

### PP activities in the stages of the funding process

In recent years several HFs have taken action within their organisations regarding PP: in the timespan from conducting the questionnaire (2014) to conducting the interviews (November 2015–March 2016) HFs have started new PP initiatives and/or professionalised existing PP activities. Most Dutch HFs have chosen to carry out PP activities in more than one stage of the funding process (overview: Fig. 2*, examples:* Table 1). Stage 1 is used by ten out of nineteen HFs for PP activities, stage 2 by eight HFs and stage 3 by fourteen HFs.

**Table 1:** Examples of PP per stage

*Example of PP in stage 1*	
In focus groups with patients, a list of possible research topics is created. These research topics are presented online to the grassroots constituency of the HF so they can indicate their personal priority preferences. The prioritised research agenda is aligned with research agendas drafted by clinicians and researchers. Only research that falls within the top 5 research topics is funded.	
*Examples of PP in stage 2*	
Example I	
A letter of recommendation from a patient organisation or other organised patient group needs to be included when submitting the research proposal. A deadline for contacting this patient group is added.	
Example II	
It is mandatory for the researchers or research institute to contact an organised patient group and ask them to rate their research proposal, before submitting it to the HF. The proposal will be rated on two topics: relevance of the topic and the extent to which they were involved and listened to in preparing the research proposal. The rating results need to be included when submitting the research proposal.	
*Example of PP in stage 3*	
Patients meet up to discuss a research proposal, because when reviewing only individually without a discussion, a good formulation of arguments might not be achieved. This may lead to unclear reasoning for the final judgment and decision on the proposal.	

#### Stage 1, strategic decision-making about the focus of research

Before setting out a call for research proposals, most HFs decide on a direction or focus that researchers should adhere to in their research proposals. In deciding this direction, approximately half of the HFs specifically include the perspectives of patients. These HFs gave two reasons for including patients specifically in this stage. It justifies research-funding decisions to their grassroots support. And research conducted in a direction chosen by patients is relevant by definition. A reason *not* to include patients in this stage is that there are too many diseases that fit the scope of the HF and therefore multiple research agendas would have to be generated, which was seen as an unattainable goal. Another argument was that the relevance of research based on the perspective of patients and other stakeholders was preferably addressed in other stages of the funding process.

##### Participation activities in stage 1

The main method used to decide on a direction of research with the input of patients is producing a research agenda that is co-drafted by patients. HFs generally have multiple research agendas drafted with separate focus groups consisting of patients, healthcare professionals, family caregivers and researchers. Subsequently, they are combined into a final shared research agenda which is expected to be broadly supported by all the stakeholders involved. Successful examples include the research agendas for burn survivors, people with respiratory diseases and heart diseases [9, 20, 21].

HFs may *combine research agendas from different stakeholder groups* into one final shared research agenda themselves, or this may be done during a joint discussion between the different stakeholders.

*Prioritisation of the topics* on the research agenda is carried out either within the focus groups identifying the research topics or in joint discussion rounds or individually on paper. Some HFs request prioritising input from a bigger group than those who contributed to the research agenda. They conduct online questionnaires or polls, allowing grassroots supporters the opportunity to prioritise the identified research topics.

#### Stage 2, call for and receipt of research proposals

Seven HFs have stated that they consider it their responsibility that researchers make involvement of patients a priority when submitting research proposals for funding. This involvement starts at drafting the research proposal. HFs have articulated criteria which have to be met by researchers applying for funds. This can be that PP is an absolute condition for research proposals to be submitted, or -more often- stating that patient involvement is reviewed by the referees or advisory boards and thus an important part of the decision-making process. In receiving the proposals the HFs do not perform an immediate check whether or not PP has taken place. The HFs who do review patient involvement, do so later on in stage 3. Three reasons are mentioned for participation in this stage. First, it is a good reflection of the importance and value that the HF grants to PP. Second, it gives researchers, HFs and decision-makers in stage 3 insight into the relevance of the proposed research for patients. Lastly, it can help make PP a normal procedure instead of a rarity; it ‘orders’ researchers to actively work with patients. In the HFs’ experience, this often led to researchers experiencing the importance of PP in general and, perhaps more importantly, the added value that patients can give to their research projects. No specific reasons were mentioned for *not* carrying out PP activities in this stage.

##### Participation activities in stage 2

PP in preparing the research proposal is rarely an *absolute condition* set by HFs for funding. How researchers should involve patients is not always pre-defined by the HFs either. In that case, HFs only ask researchers to state if and how patients (or patient representatives) have been involved during the preparation of the research proposal. HFs may want to consider how patients’ input has changed the proposal. Because of the lack of guidelines, this often resulted in short and incomplete answers, or PP was not done at all as no patients could be found.

When a *clear guideline is provided*, it improves insight into the degree of input by patients and ensures that the patient input is valuable instead of a formality. Some HFs also provide a time frame, including deadlines for contacting patients, because involving patients early on improves the quality of the research proposals and allows patients more time to respond.

#### Stage 3, decision-making about the funding of specific research proposals

Traditionally, submitted research proposals were reviewed by external expert referees and scientific advisory boards, consisting mainly of scientists and doctors. Most HFs find the approach of peer-review without patients outdated, and fifteen HFs include patient reviewers, mainly to gain insight into the relevance of the research proposals for patients. Several HFs are currently in the process of setting up a system which involves patients reviewing research proposals. HFs that have no intention to do so, explained that they have involved patients in the first or second stage of the funding process and thus already take their views regarding the relevance of the research into consideration.

##### Participation activities in stage 3

In almost all (15) HFs, patients *co-review research proposals*, either as an external referee or as a member of a patient/societal committee. Many HFs have established a *patient panel or a patient advisory committee* to co-decide or co-advise (differs per HF) which research finally gets funded. Moreover, there are HFs that have set up a societal advisory board consisting of patients, healthcare professionals, family caregivers and/or citizens. The number of patients in any of these settings ranges between three and twelve.

Patients can co-review the research proposal, but often they are given a lay summary. Lay summaries help the patients understand medically or technically complicated proposals and the scientific jargon used. Some patients when given the original research proposal focussed on technical, scientific details which do not fall within the scope of their review responsibilities, but rather to the responsibility of the scientific reviewers. There are various ways to review the proposals. Several HFs let patients individually review the proposal on paper, sometimes followed by a meeting where patients come together to discuss the proposal, clarify their opinion and form a consensus regarding the research proposal. Other HFs conduct the entire patient-review process through a discussion, with the HF’s project leader or a researcher as facilitator. The facilitator presents the research proposal, explains if and how the research has societal relevance, and answers patients’ questions. The goal of these sessions is to come to a decision regarding the research proposals, agreed upon by all participating patients, and to give feedback to researchers.

The review criteria patients receive differ according to HF. For most HFs, the criteria must include the (1) relevance of the topic, for patients and for society as a whole; (2) burden for the study subjects; and (3) feasibility of the research. Only a few HFs have prepared the criteria in cooperation with patients.

Finally, if and to what extent training or coaching is provided for the patients to help them through the reviewing process differs per funding foundation, ranging from no training to several days of training.

Often, the balance between the voice of the patients versus the voice of the scientific advisory board and reviewers has not been precisely established and no official policy documents regarding this balance were found. Some HFs consider the patients’ decision as input for further deliberations by a scientific committee. Other HFs have members of the scientific committee deliberate with patients (a delegation), comparing the judgments of both groups, in order to come to a final consensus. In that case, it is possible that no or only one patient is present when the final decision is taken. Because of the lack of policy documents, the actual influence of patients’ advice as opposed to the professionals’ advice cannot be deduced and often remains unclear.

### Main challenges and possible solutions

HFs encounter various challenges in their PP activities. The three main issues experienced are: *how to accommodate diversity; to what extent should patients receive training*; and *who is responsible for patient-researcher dialogues.* Solutions proposed by participants of the dialogue meeting are shown in Table 2, these solutions are based on their professional experience.

**Table 2:** *Proposed solutions to main challenges*

*Proposed solutions to accommodate diversity*	
- Diseases could be combined under broader disease domains when dealing with a patient body with a large diversity of diseases or disease types.	
- Family caregivers or entrusted physicians can participate as patient advocates when participation is not possible for certain patient groups due to their disability, or when no patients willing to participate can be found because the prevalence of the disease is low.	
- A participant profile can be made, including what skills are needed for specific PP activities or tasks.	
- A database of patients interested in participating in the funding process can be set up, including their background, skills and interests in order to link them to the above-mentioned profiles. Patients with different skills, background, educational level can join different PP activities.	
- Training can be organised for patients who are willing to participate, before deciding whether or not they are capable of participating in certain activities.	
- Cooperation can be established with local organisations that work with target groups which are harder to reach (e.g. ethnic minorities, people with low SES).	
*Proposed solutions for patient training*	
- A ‘chief listening officer’ can be appointed, to listen to the participatory wishes and needs of patients and to give an overview of bottlenecks experienced regarding the prerequisites for effective participation.	
- It can be acknowledged that there is a limit to the willingness and capacity or workload of patients, and a central point (or person) can be set up where patients can indicate their capacity (changed) or manageable workload.	
- An incentive can be provided in the form of a reward for researchers who commit to establish a dialogue with patients (on their own initiative and in their own way).	
- Contact days for groups of researchers and patients can be facilitated, in order to bridge the gap between them.	
- Training days for researchers can be facilitated, with possibly patients providing training sessions on ‘how to meaningfully interact with patients’.	
- A HF employee can be appointed to answer questions from researchers on how to contact and have a successful dialogue with patients.	

#### How to accommodate diversity?

An important challenge which is encountered by most HFs revolves around the question: ‘who should participate’. This challenge is encountered in stage 1 (strategic decision-making about the focus of research) and 3 (decision-making about the funding of specific research proposals). Most HFs relate to a diverse patient group, consisting of people with different diseases, social economic status (SES), ethnic minorities, and verbal and cognitive capabilities. All HFs state that it is a challenge to include a group of patients, doing justice to this diversity and without leaving out an important target population of the HFs.

‘Suitable’ patients have to be recruited, who are ‘capable’ of participating in the decision-making process. Suitable was explained as being able to transcend individual or personal needs, i.e. participating patients are able to think beyond the boundaries of their own disease and thus can speak for a broader patient group. Capable was explained as having the ability to reason with others concerning complex topics and to learn, reflect and make decisions. HFs doubt whether they should only include patients with a higher educational level, although this would not be a fair representation of the patient body since a large group would be excluded, often the ones most in need.

#### To what extent should patients receive training?

Several HFs struggle with whether or not patients should receive training and what it should consist of. This challenge is encountered in all three stages. The training should provide patients with enough information to make participation meaningful, while not professionalising them so they lose their unique perspective as a patient. HFs stress that when deciding on training and its content, it will involve making additional demands of the patients. Therefore, it is an important challenge to monitor the patients’ capacity and willingness to handle the workload.

#### Who is responsible for patient-researcher dialogues?

HFs question who is responsible for a successful dialogue and what skills the researchers need to establish a dialogue. This challenge is encountered in stage 1 (strategic decision-making about the focus of research) and 2 (call for and receipt of research proposals). HFs point out that it is sometimes difficult to decide where their responsibility ends and that of others, in this case researchers or research institutes, begins. They find that researchers often do not yet see the added value of PP or know how to accommodate PP, and therefore the HFs take it upon themselves to ensure that dialogues are established between patients and researchers. These are dialogues in stage 1, for example setting a research agenda with both researchers and patients, but also in stage 2 where researchers should work on their research proposal in cooperation with patients. They want to find effective ways to ‘persuade’ researchers to take on the responsibility of setting up a dialogue with the patients themselves and get the necessary training.

## Discussion

The HFs agree that the time of questioning whether or not patients should be involved in the process of allocating funds to health-related research is over. The focus is now on *how* to organise, optimise and entrench PP meaningfully in the HFs’ funding processes. This process is being given active support by the Dutch government; policy documents underline the need for so called responsive research, so funding will be allocated to research with societal relevance [22]. Furthermore, a health-related research funding organisation (ZonMW), funded by the Dutch government, also works with patient panels for the different calls for proposals they set out. Dutch patient advocacy groups are collaborating to combine their influence and together make recommendations for the successful involvement of patients in health-related research [16]. Similar participatory initiatives, supported by governments, are seen internationally. Exemplary is the government of the UK who funded ‘INVOLVE’, a national advisory group which aims to support active public (including patient) involvement in the healthcare system, public health and social care research [23].

All Dutch HFs are involving patients in at least one stage of their funding process. From the UK-based study of O’Donnell and Entwistle, we see that stages 1 and 3 are chosen as the focal points for PP activities [18]). UK activities include postal surveys or focus groups, consumer panels, and consultation meetings with consumer representatives from voluntary organisations to identify and prioritise important research topics [18]. In the Netherlands, stage 1 is used for PP by 10 out of 19 HFs; drafting research agendas is quite common, postal/online surveys are carried out less often but is increasingly seen as a method with high potential.

Although only 8 of 19 Dutch HFs have PP activities in stage 2 and those activities are often less structured or in a pilot phase, most HFs voiced their intention to involve patients here in the future. If researchers include patients before submitting their research proposal, they will experience that the research proposal is improved, facilitating possible further cooperation [24].

Stage 3 is most often chosen for PP activities in the Netherlands. Approaches to involve public/patients include co-reviewing research proposals and being a member of a review committee [18]. This corresponds to the situation amongst the HFs: most of them have set-up a system where patients co-review the proposal and do so with similar review criteria. This is also is what is to be expected of HFs, who’s main task is to make funding decisions on research proposals which ultimately should benefit the patient. However, a UK-based study of ten research funding bodies by van Bekkum and Hilton showed that even though most funders find stage 3 promising for participation, only two of them involved the public in a committee and just one included them in their external review [8]. Some UK funders feared the public’s opinion might be biased by emotions or a personal agenda. An Australian-based study of PP activities of a funding organisation for cancer research also considered stage 3 as promising for participation. They set up a patient (consumer) panel to review research proposals based on criteria of the public value of the research [3, 4].

Concerns were voiced by the Dutch HFs but also in the UK and Australia regarding the identification of ‘appropriate’ participants and difficulties with recruitment and deciding who should represent patient and public perspectives [17, 18]. The Dutch HFs specifically perceived a difficulty in reaching certain patient groups, due to cultural or linguistic barriers and the fact that these groups are also underrepresented in patient organisations, while the cultural diversity of the Dutch society and thus of the patient population is growing [25]. Similarly, in two UK studies, funders noted that people on committees tended to be better educated, such as retired academics or professionals, and wondered if they represent the entire patient body [8, 18]. This was also voiced by Wind, who stated that she wants to get rid of the phenomenon that mainly the ‘intellectual elite’ participate, and insisted that a broader patient population should be included [26]. The authors recognise that for each stage the ‘appropriate participants’ are different. Stage 1 would be the stage where patients with a wide range of social, economic and educational status can participate in various participatory methods, while stage 3 is more prone to the so-called ‘intellectual elite’ where patients’ representative participate.

A second prominent issue of concern is whether or not patients should receive training before they participate and what this training should consist of. Many Dutch HFs raised this issue, and it was extensively discussed during the workshop. Those in favour of training pointed out that to participate in stage 3, participants needed insight into scientific research and the funding process to be able to make a good judgment as well as to become a helpful discussion partner for the scientific review committee and researchers. The opposite opinion entailed a worry that patients become ‘protoprofessionals’ and lose their unique patient perspective. Especially when participating in stage 1, a training was not deemed necessary. It is remarkable that in UK and Australian studies, training is provided for patients but no concerns were raised [3, 4, 8, 17, 18]. However, in literature we see academics who are and who are not in favour of the training of patients. In the UK a research awareness training programme has been developed for patients, in order for them to actively and meaningfully be involved in research, evaluations and audit projects [27]. According to Dedding and Slager [28], training is not necessary but using more creative methods might help to accommodate non-academic participants and encourage them to share their knowledge, experiences and needs [28].

The third challenge that was often mentioned by the HFs concerns the responsibility for patient-researcher dialogues. This mainly regards the dialogue needed in stage 1, for example while setting up a shared research agenda, as well as in stage 2 where researchers should work together with patients on their research proposal. HFs deem themselves, together with researchers, responsible for the establishment of this dialogue and cooperation. However, they find that in practice this task often solely falls upon them. HFs take this responsibility and facilitate contact between researchers and patients, however they hope that in the near future this will become a shared responsibility.

### Limitations of the study

Several HFs have a group of people working on PP, while only one of them was interviewed per HF. It might be that other interviewees from the same HF would have given a different line of argumentation or had different experiences. The position of the interviewees within a HF varied. This might have caused a difference in the information provided: for example, a director might have different arguments and explanations regarding PP activities than the employees who carry out these activities in practice.

No patients were involved in the questionnaires and interviews, because solely HF employees were asked to participate. However patient advocates joined the PP advisory team who advised in the set-up of this study. Furthermore, three patient organisations participated in the workshop.

In recent years, many PP initiatives have been set-up within the field of health-related research. We included non-scientific documentation (policy documents of the UK and Dutch governments and from patient advocacy groups) on these initiatives. More grey, non-scientific literature is published regarding PP initiatives than these examples. Although of importance, including these is outside the scope of this study.

## Conclusion

Most Dutch HFs are committed to PP, but further broadening and optimising of patient involvement is still needed, and transparency and cooperation between funding foundations are necessary. Our proposed solutions to the experienced challenges during PP activities, could serve as inspiration for national and international foundations that want to include patients structurally in their funding process.

## Data Availability

The datasets generated and/or analysed during the current study are not publicly available, since this data contains transcripts of interviews in which specific opinions of Health Fund employees are included, but are available from the corresponding author on reasonable request.

## Abbreviations

HF: health funds, health-related research funding organisations
PP: patient participation
SGF: Collaborative Health Funds (Samenwerkende Gezondheidsfondsen)
